# The Development of a Sub-Surface Monitoring System for Organic Contamination in Soils and Groundwater

**DOI:** 10.1100/tsw.2002.203

**Published:** 2002-04-24

**Authors:** Sharon L. Huntley, Lawrence J. Ritchie, Steven J. Setford, Selwayan Saini

**Affiliations:** Cranfield Centre for Analytical Science, IBST, Cranfield University at Silsoe, Silsoe, Bedfordshire MK45 4DT, UK

**Keywords:** organic, contaminants, hydrocarbons, hydrocarbon contamination, fuel, benzene, toluene, ethylbenzene, xylene (BTEX), methyl tert-butyl ether (MTBE), ethyl tert-butyl ether (ETBE), near infra-red sensors, sub-surface sensors, data logging, groundwater pollution, water, pollution, immunoassays, landfill, contaminated land, environmental monitoring, environmental analysis, early warning systems

## Abstract

A major problem when dealing with environmental contamination is the early detection and subsequent surveillance of the contamination. This paper describes the potential of sub-surface sensor technology for the early detection of organic contaminants in contaminated soils, sediments, and landfill sites. Rugged, low-power hydrocarbon sensors have been developed, along with a data-logging system, for the early detection of phase hydrocarbons in soil. Through laboratory-based evaluation, the ability of this system to monitor organic contamination in water-based systems is being evaluated. When used in conjunction with specific immunoassays, this can provide a sensitive and low-cost solution for long-term monitoring and analysis, applicable to a wide range of field applications.

## INTRODUCTION

Land-based contamination from organic pollutants has been identified as a major potential risk to water resources throughout Europe, particularly from the more mobile BTEX compounds (benzene, toluene, ethylbenzene, and xylene) and oxygenated fuel additives, such as methyl *tert*-

butyl ether (MTBE) and ethyl *tert*-butyl ether (ETBE). Large volumes of contaminants leaking into soils are likely to migrate down to the water table[1]. The European WATCH programme (Water Catchment Areas: Tools for Management and Control of Hazardous Compounds) is a Framework V RTD programme for the protection of European ground water and drinking water resources[2]. As with other major European Commission programmes (for instance, the Concerted Action networks CLARINET and NICOLE), the WATCH programme has recognised the urgent need for early warning monitoring systems to detect ground water pollution incidents. Through the development of environmental monitoring systems and sustainable management, the WATCH programme aims to facilitate the implementation of existing and proposed Water Directives.

One approach is the development of low cost and robust sub-surface sensors that can act as early detection systems for organic pollutants. When used in conjunction with suitable BTEX immunoassays (total petroleum hydrocarbons, PAHs, BTEX, MTBE, etc.), the sensors can offer a means of continuous pollution surveillance for both vulnerable and contaminated sites, with rapid *in situ* analysis of the specific contaminants.

## EXPERIMENTAL DESIGN AND PROCEDURES

### Sensor Design

The sensor consists of two main parts: the sensing element in the head and the sensor body containing the electronic circuitry. The sensor body is ruggedly manufactured in-house from tubular brass, 16 mm in diameter, with a total length of 100 mm. The novel design of the sensor head (Fig. 1) incorporates a highly reflective hydrocarbon-specific membrane[3], supplied by Pall-Gelman Ltd (Portsmouth, U.K.). The membrane is protected on the exterior face by a 1-mm aluminium mesh. To prevent any reflectance from the mesh, it is anodised to give a non-reflecting black surface. A 1-mm oil-resistant rubber spacer separates the membrane from the sealed glass surface of the sensor body housing the electronic circuitry.

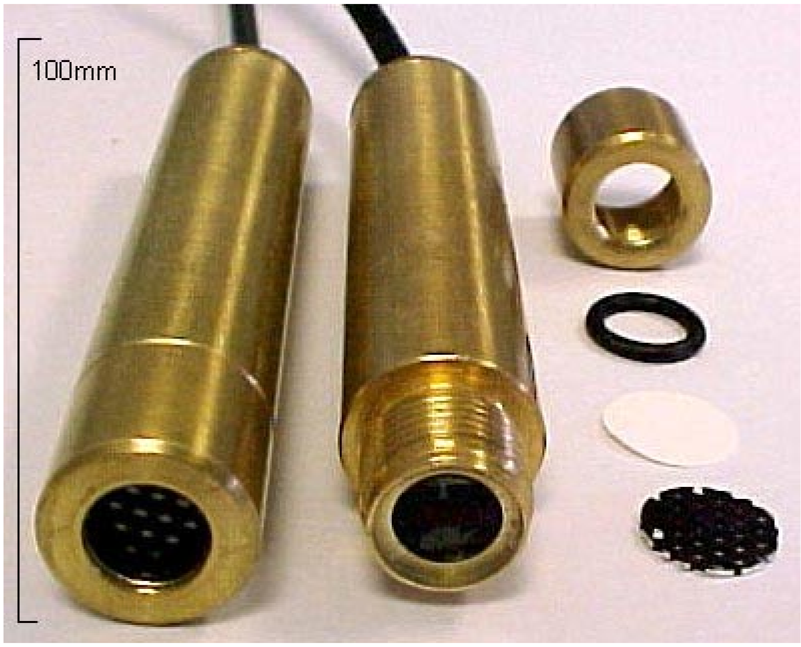

**FIGURE 1.** The sub-surface fuel sensor. The sensor on the right shows an exploded view of the sensor head assembly, exposing the NIR photoemitter/detector situated behind the glass face of the sensor body.

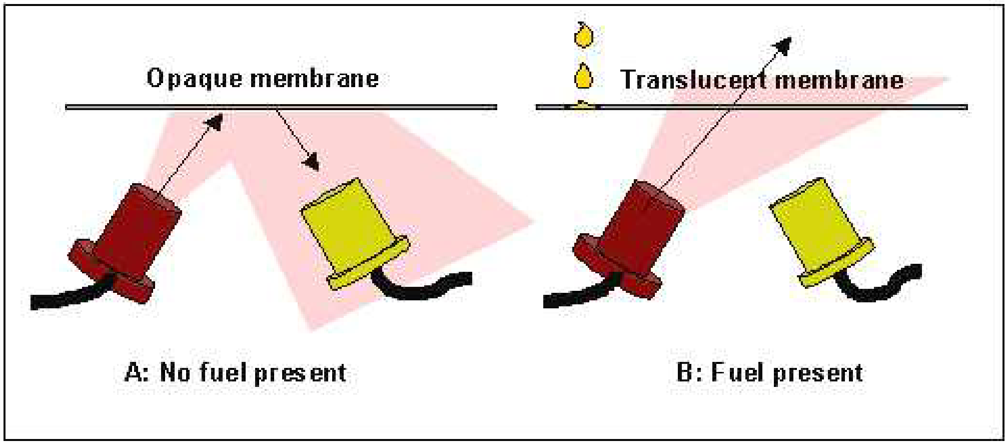

**FIGURE 2.** (A) Opaque membrane provides high reflectance and a low signal; (B) Absorbed fuel results in a translucent membrane, which results in low reflectance and high signal.

## Electronics

Using simple electronic circuitry, a rechargeable battery powers a matched emitter/detector pair consisting of a near-infrared (NIR) light-emitting diode (950 nm) adjacent to a phototransistor detector built into the sensor body. Under normal operating conditions, the light is reflected back from the membrane to the phototransistor. The amount of light received by the phototransistor is monitored as a baseline voltage at the collector electrode. When the membrane comes into contact with hydrocarbon fluid, the membrane turns translucent, allowing the majority of the light to be transmitted (Fig. 2). The amount of reflected light decreases, resulting in an increase in the measured output voltage. Each sensor has been designed to draw a maximum of 10 mA in order to prolong battery life and provide long-term *in-situ* monitoring at remote locations. In the absence of hydrocarbons, reflectance measured at the assembled sensor head provides a baseline measurement of approximately 2 V. An increase in voltage (up to a maximum of 4.8 V) indicates poor reflectance as a result of oil absorbed by the membrane.

Multiple sensors can be linked as a distributed system, connected to a central on-site data-logging device, and used to monitor large areas, e.g., around underground fuel storage tanks or deployed across a susceptible site in a grid-type matrix. Each sensor is equipped with 30 m of insulated cable and equipped with a standard RS232 serial interface 9-pin connector.

## Data Acquisition and Transmission

A multiple parameter data logger has been modified to record the analogue signals from multiple sensors via standard 9-pin connections (Fig. 3). The mobile monitoring unit consists of the combined sensor interface, data logger, wireless Internet communications, and control microelectronics with a rechargeable 8-volt battery supply[4]. The system has been developed with the potential for an unlimited number of sensors to be configured together. In its current configuration, the unit can operate with 8 networked sensors for approximately 6 months. The sensor-based system has been specifically designed to enable low-cost wireless data acquisition in unattended, remote locations. Collected data is stored in the unit’s memory (RAM) along with the date and time of sampling.

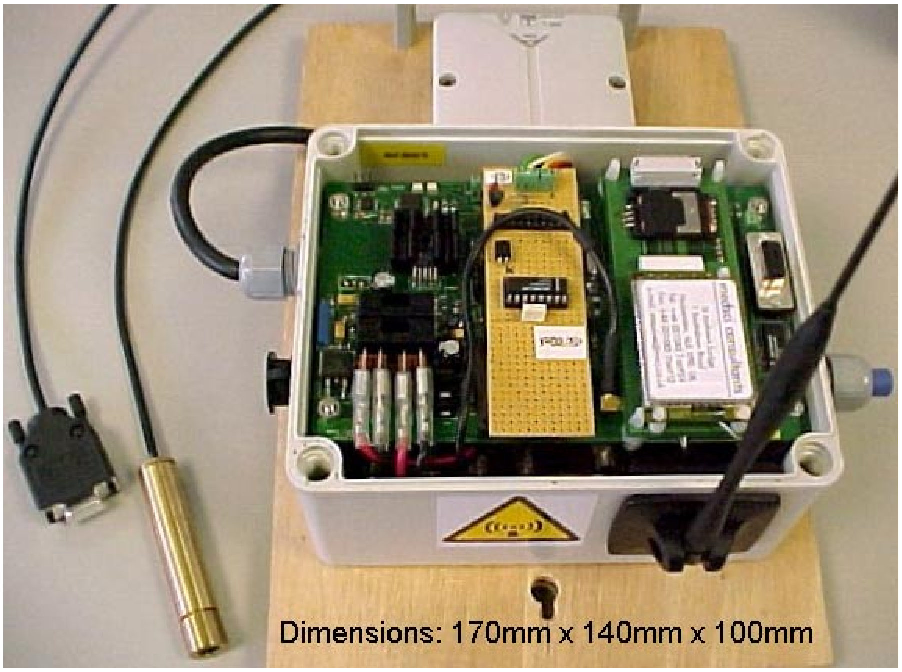

**FIGURE 3.** The field-based monitoring system consists of the data logging unit, to which *n*-sub-surface sensors may be connected via standard 9-pin sub-D connectors.

Data can be delivered periodically to the user via Internet communications and digital telephone networks using Short Messaging Service (SMS) notifications. Using an e-mail client application, e.g., a program such as Microsoft Outlook Express, the unit makes a cellular telephone call and connects to the Internet. The accumulated data is then sent as an e-mail using standard Internet protocols to one or more host e-mail addresses. The data is received as a binary file attached to the email. Using a simple executable programme, the data is extracted and can be read into other programs (e.g., Microsoft Excel) for further analysis. Further versatility of this monitoring system is that the sampling and data notification rates may be reprogrammed via the Internet. Additionally, data may be retrieved on site at any time using a portable computer.

## INTEGRATION OF THE NIR SENSORS WITH IMMUNO-DIAGNOSTIC TOOLS

The NIR sensors will respond to general hydrocarbon populations in soil and sediments. Therefore, in order to provide a more detailed initial field-based characterisation of a contamination problem, triggering of the IR sensors will be followed by the use of immunoassay-based methods for the specific characterisation of key target analytes within the vicinity of the triggered device(s).

Immunoassays are achieving ever-greater credibility for field-based site characterisation, as evidenced by their acceptance by the EPA as a valid analytical screening tool. For example, the EPA SW-846 test methods recognise (commercially available) immunoassays for screening of petroleum hydrocarbons (#4030) and polynuclear aromatic hydrocarbons (#4035) in soil[5]. The tests are rapid, sensitive, low-cost, and simple, allowing field-based usage.

## PRELIMINARY EXPERIMENTAL PROCEDURES AND AIMS

Initially designed for soil and sediment monitoring, the sensors have been shown to respond to phase hydrocarbon contamination[3,6]. Recognising the potential risk to European water resources, determining the response to a range of hydrocarbons in water may enable initial quantification for pollution incidents. For laboratory-based analysis, the sensors were connected to an 11-channel 10-bit ADC-11 parallel port analogue to digital converter from Pico Technology (Cambridge, U.K.) via an in-house signal conditioning board. A data acquisition programme was written using LabView (National Instruments Corporation, Newbury, U.K.) to enable real-time data collection, analysis, and display. Acquired data was then exported and analysed using a standard spreadsheet programme.

In order to test the sensor response to fuels suspended in water, standard grades of unleaded petrol, diesel, and laboratory grade MTBE were used to evaluate the sensitivity of the membrane and the corresponding signal response. For each fuel evaluated, the percentage of fuel in a fuel/water mixture was varied from 100% fuel down to 1% fuel in water, in steps of 10%. Due to the immiscibility of petrol and diesel in water, samples were prepared by shearing measured mixtures of fuel and water to create a suspension, with measurements performed prior to phase separation.

## INITIAL RESULTS AND DISCUSSION

As a result of early investigations within this project, the sensor head has been redesigned for improved functionality and performance in both soil and water matrices. Preliminary results for monitoring water contamination with the redesigned sensor have been encouraging. Whilst different in chemical composition, all three fuels tested have been shown to be rapidly absorbed by the membrane. Results from 20% water contamination from both petrol and diesel are shown in Fig. 4. Rapid sensor triggering (with response over 4 V) occurred for all spiked samples down

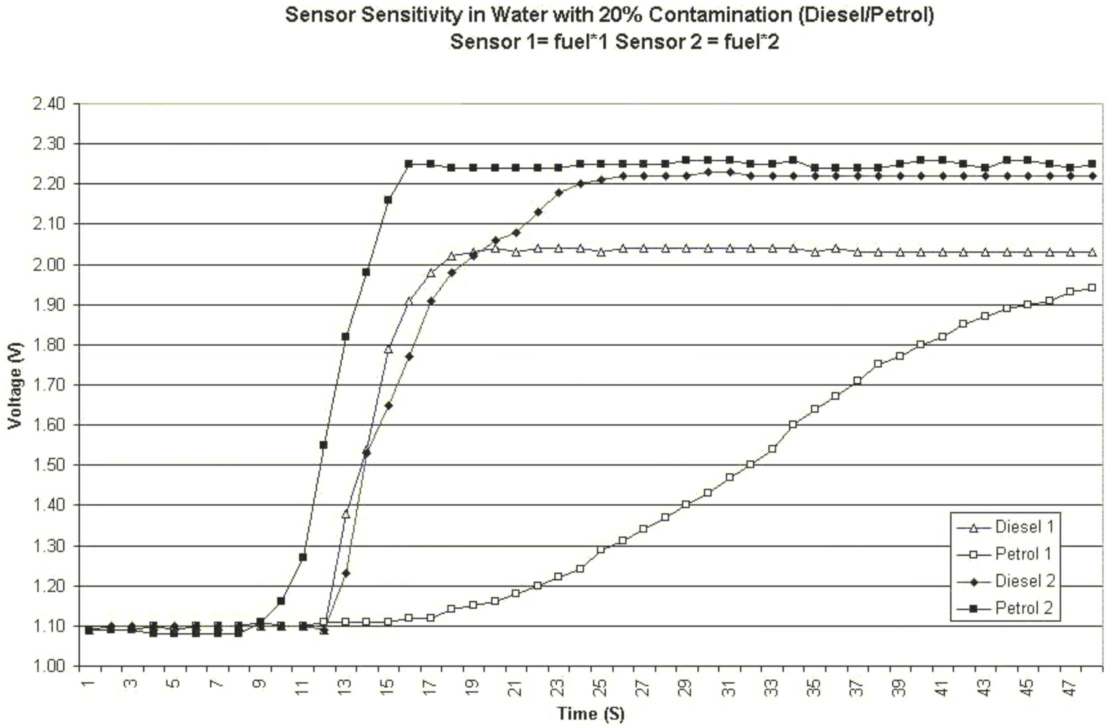

**FIGURE 4.** Sensor response to 20% free-phase fuels in water.

to 10% contamination in water. Sensor responses were inconsistent for water samples contaminated at lower levels. With free-phase MTBE, once the sensor had triggered, a loss of signal was detected. This was attributed to the high volatility of the compound. It was observed that rapid evaporation of the compound from the membrane surface resulted in corresponding signal loss. No apparent residual effects on the membrane surface were detected and the signal returned to the initial baseline value. When a further MTBE sample was tested using the same membrane, the sensor response was equal to that of previous investigations.

## CONCLUSIONS

A simple, low-cost, robust multi-sensor system with associated data logging/transmission for the early detection of aliphatic hydrocarbon pollution in soils and sediments has been described. Laboratory-based assessments will continue in order to achieve an optimal response to lower concentrations of biphasic contamination in both soil and water matrices. Identification and quantification of specific contaminants can be achieved using on-site immunoassay test kits/sensors combined with laboratory GC/MS. Work in progress includes the development of antibodies and corresponding immunoassay test kits/simple immunosensor devices for the quantitative measurement of the fuel oxygenates MTBE and ETBE in soil and water samples. The goal of this work is to provide an integrated early warning/characterisation system for protection of groundwater at susceptible sites. This screening approach will be supplemented by more detailed laboratory-based evaluation/validation procedures as appropriate. An overall reduction for on-site monitoring and characterisation costs is envisaged.

## ACKNOWLEDGEMENTS

This paper was presented at the CSIC/ESF workshop "Analysis, Toxicity, and Biodegradation of Organic Pollutants in Groundwater from Contaminated Land, Landfills, and Sediments." Barcelona, Spain. URL: http://www.cid.csic.es.

The project is supported by the European Commission (EVK1-CT-2000-00059) under the WATCH programme (Water Catchment Areas: Tools for Management and Control of Hazardous Compounds). URL: http://www.watch-eu.org.

The data-logging device has been developed in conjunction with Medsci Consultants Limited, Publication PUB001. Medsci Consultants Ltd., St. Andrew's Lodge, 7 Southdown Road, Harpenden, Hertfordshire, AL5 1PD, U.K. E-mail: enquiries@msci.co.uk.
